# Early Alzheimer's diagnosis: U.S. primary care physicians and use of blood biomarkers

**DOI:** 10.1002/alz.70986

**Published:** 2026-01-18

**Authors:** Jeffrey M. Burns, Susan Alford, Justine Coppinger, Martí Jiménez‐Mausbach, Sutapa Ray, Hemant Pandey, Rosemary Laird

**Affiliations:** ^1^ Department of Neurology KU Alzheimer's Disease Research Center University of Kansas Medical Center Fairway Kansas USA; ^2^ Novo Nordisk Inc. Plainsboro New Jersey USA; ^3^ C2N Diagnostics, LLC Saint Louis Missouri USA; ^4^ Novo Nordisk A/S Soeborg Denmark; ^5^ Brain and Spine Center Chandler Arizona USA; ^6^ Mymemoryclinic.org Melbourne Florida USA; ^7^ NAN Navigator Inc. Orlando Florida USA

**Keywords:** Alzheimer's disease, Alzheimer's disease diagnosis, blood biomarker tests, blood biomarkers, cognitive decline, cognitive impairment, diagnosis, diagnostic workflow, general practitioners, plasma biomarkers, primary care physicians

## Abstract

**INTRODUCTION:**

We aimed to explore primary care physicians’ (PCP) attitudes, perceptions, and barriers toward Alzheimer's disease (AD) diagnosis and incorporating blood biomarker (BBM) tests into the diagnostic workflow.

**METHODS:**

Remote 60‐min interviews with 20 PCPs were conducted (May 2023). Participants included generalists and geriatricians representing urban, suburban, and rural U.S. practices. Interviews encompassed early AD diagnosis, PCP role, and BBM test implementation.

**RESULTS:**

Most PCPs view investigating cognitive decline as an important part of their role and are somewhat confident in diagnosing AD. Barriers include the complexity and inefficiency of current diagnostic workflows, lack of effective treatments, and stigma. PCPs consider BBM tests accurate and cost‐effective but have concerns about reimbursement and diagnostic pathway placement.

**DISCUSSION:**

PCPs are interested in AD diagnosis and receptive toward BBM testing. Education on BBM test use and AD diagnosis may benefit PCPs in the care of individuals with cognitive decline.

**Highlights:**

- Early Alzheimer's disease (AD) diagnosis is crucial for initiating treatment
- Primary care physicians (PCPs) find investigation of cognitive decline important
- PCPs consider blood biomarker (BBM) tests accurate and cost‐effective
- PCPs seek clarity on reimbursement of BBM tests and their context of use
- Education on BBM test interpretation and AD diagnosis may benefit primary care

## 1 INTRODUCTION

Blood biomarker (BBM) assays are emerging as important tools to provide a biological determination of presence or absence of Alzheimer's disease (AD) pathology and may become more easily accessible in primary care than traditional AD diagnostic tools, such as positron emission tomography (PET) imaging and cerebrospinal fluid (CSF) biomarker analysis.^1^ Advances in detecting specific AD biomarkers in blood are being used to develop accurate and robust tests that can aid in the diagnosis of AD. The sensitivity and specificity of these tests in detecting amyloid pathology ranges from 80% to 97%, depending on the technology, test model (binary tests or those with an intermediate category), individuals tested, and combination of BBMs used.^2^, ^3^, ^4^, ^5^, ^6^, ^7^, ^8^, ^9^

The emergence of BBM diagnostic tests for AD will allow for a more precise diagnosis, moving away from a reliance on clinical symptoms, and with the potential to substantially advance clinical care.^1^, ^10^ Debate about the role of biomarkers and clinical symptoms in diagnosis is ongoing. According to the 2024 Alzheimer's Association (AA) Workgroup's diagnostic criteria,^1^ detection of an abnormal Core 1 biomarker using an accurate BBM assay (specifically phosphorylated tau‐217 [p‐tau217] or the hybrid ratio p‐tau217/np‐tau217, represented as %p‐tau217) is sufficient to establish a diagnosis of AD and inform clinical decision‐making throughout the disease continuum. In contrast, the International Working Group (IWG) proposes that a diagnosis of AD is established using a comprehensive clinical workup that includes the use of biomarkers, but avoiding diagnoses based solely on biomarkers reflective of AD pathology.^11^ Until therapies are available for preventing or delaying symptoms in individuals with preclinical AD, the use of current BBM tests is likely to remain confined to symptomatic individuals (with mild cognitive impairment [MCI] and dementia), not cognitively unimpaired individuals.

Although biomarker assessments reflecting underlying biology have shifted clinical AD diagnosis toward that of inclusion rather than that of exclusion of other causes, the diagnosis can still be experienced as slow, complex, often inaccurate, and inconsistent across health care systems.^12^, ^13^, ^14^, ^15^ The role of primary care physicians (PCPs) in AD diagnosis is gaining importance with the increasing number of individuals presenting to primary care with cognitive complaints.^16^, ^17^ PCPs have previously reported the following barriers to making AD diagnoses: the time‐consuming diagnostic workup, low confidence in making a definitive AD diagnosis, and the stigma associated with an AD diagnosis.^16^, ^18^

Known barriers to acceptance and use of BBMs in primary care include gaps in knowledge about the assays and their accuracy for detecting AD pathology as well as lack of skills to interpret them. In addition, the full clinical value of an early and precise diagnosis is proportional to the efficacy and safety of available AD therapies.^19^, ^20^, ^21^, ^22^ Thus, the advent of anti‐amyloid therapies has increased the potential utility of BBM tests in practice.

Through in‐depth PCP interviews across the United States, our aim was to investigate PCPs’ attitudes on and perceptions of early AD diagnosis, their perceived role in diagnosing AD, as well as their views on the challenges and barriers to adopting BBM tests as part of the diagnostic workflow. An infographic and a lay language summary of this study can be found in the **Supporting Information**.

## 2 MATERIALS AND METHODS

### 2.1 Study design

An exploratory study consisting of 20 in‐depth 60‐min interviews with PCPs took place between May 12 and 26, 2023. The interviews were conducted remotely using a screen‐sharing platform by the medical market research company 2055 Insights & Strategies.

**Research in context**

1. **Systematic review**: The authors searched PubMed for relevant publications and also applied their knowledge of initiatives and frameworks in the field to identify relevant information.
2. **Interpretation**: Primary care physicians (PCPs) report that investigation of cognitive decline is an important part of their role. Our study highlights PCPs’ receptivity toward blood biomarker (BBM) tests for improving early Alzheimer's disease (AD) diagnosis in their practices. Integration of BBM testing into the diagnostic workflow holds significant promise for earlier AD diagnosis, enabling access to care at an earlier stage of the disease.
3. **Future directions**: Continued medical education on accurate AD diagnosis, based on pathobiology and clinical presentation alongside interpretation and communication of BBM test results, may be beneficial for primary care involvement in AD diagnosis. Addressing concerns about reimbursement and ensuring the affordability and accessibility of BBM tests will be crucial for their successful implementation.

### 2.2 Participants

The participating PCPs were family or general practitioners or internal medicine physicians and recruited from a target list identified in the clinical research provider IQVIA's longitudinal prescription (LRx) and medical claims (Dx) databases between November 2019 and December 2022. The target list for this study was generated using predictive modeling based on data definition of participants and included PCPs who were predicted to treat 55‐ to 80‐year‐old individuals who were associated with at least one claim for one or more of the following predictors: memory loss/amnesia, dementia, other cognitive deficits/early stage AD, MCI/early stage AD, cognitive functions and awareness, selective serotonin reuptake inhibitor (SSRI) or neurology prescriptions, or gait and mobility abnormalities. In addition, some of the PCPs were categorized as having a geriatrician sub‐specialty if they were certified in geriatrics or care of older adults or if they self‐identified as geriatrician due to treating predominantly or exclusively individuals 65 years of age or older. PCPs were recruited across the United States from a mix of urban, suburban, and rural practice locations and could be independent or part of a hospital system or network. The participating physicians saw >30 individuals with dementia and/or suspected AD per month, of whom >10 were at early stages or with mild symptoms. All the PCPs interviewed conducted memory or cognitive assessments in their practices, such as the Mini‐Mental State Examination (MMSE), Mini‐Cog, general practitioner assessment of cognition (GPCOG), or Saint Louis University Mental Status examination (SLUMS). All PCPs gave their informed consent.

### 2.3 Data collection

**FIGURE 1:**
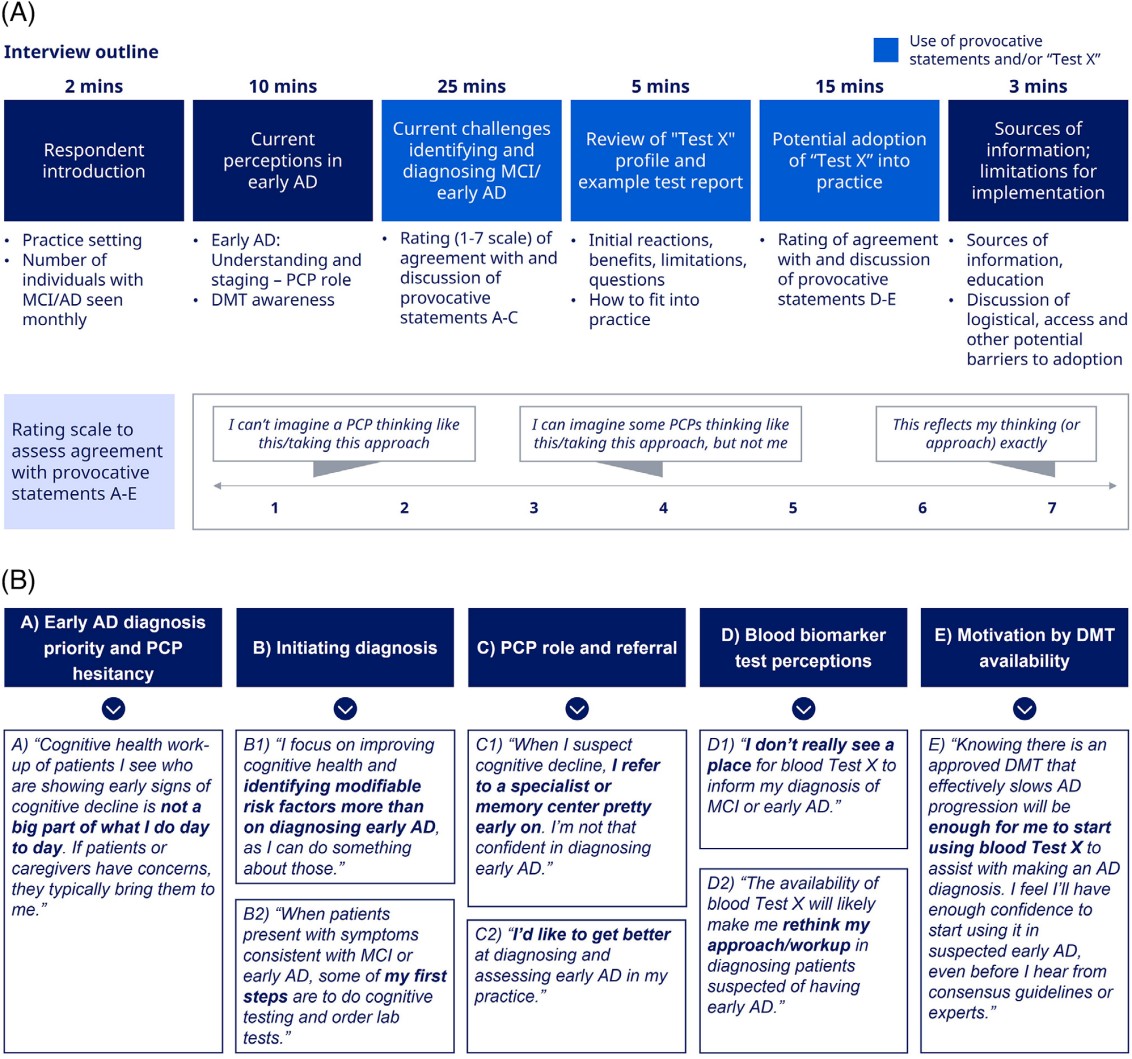
Study design. (A) Interview outline and rating scale. (B) Provocative statements. AD, Alzheimer's disease; DMT, disease‐modifying therapy; MCI, mild cognitive impairment; PCP, primary care physician.

The interviews with PCPs (outline in **Figure** **1A**) included “provocative statements” based on five overarching themes that were identified through a literature review: (A) early AD diagnosis priority and PCP hesitancy, (B) initiating diagnosis, C) PCP role and referral, (D) BBM test perceptions, and (E) motivation by disease‐modifying therapy (DMT) availability. Eight statements were formulated under these themes to foster the discussion (**Figure** **1B**). The statements were codeveloped by 2055 Insights & Strategies, C2N Diagnostics, and Novo Nordisk. The scale used for rating responses is summarized in **Figure** **1A**. A hypothetical BBM test was introduced as part of the interviews and called Test X. Characteristics of Test X can be found in **Table** **S1**. Briefly, the test measures multiple plasma analytes, including amyloid beta (Aβ) and tau peptides, using mass spectrometry. An algorithm then combines analyte ratios to give a numeric score (0–100), interpreted as either a negative (0–47) or positive (48–100) result, consistent with a negative or positive amyloid PET scan, respectively, with a sensitivity of 91% and specificity of 86%.

### 2.4 Analysis techniques

Participating physicians underwent one‐on‐one, in‐depth interviews with one of two expert medical research interviewers about their practices and perceptions regarding AD, including diagnosis, management, and treatment. Interviewers followed the same discussion guide (available as **Supporting Information**) and underwent training to ensure homogeneity in interview flow, focus, and probing. All interviews were ≈1 h in duration and were recorded.

Next, the recorded interviews underwent content analysis by a single analyst. This involved listening carefully to each interview, and capturing responses in a single document, with similar responses aggregated together and disparities highlighted. The resulting content analysis was reviewed by the interviewers, to ensure it reflected their recollections of the interviews.

The content analysis output provides an in‐depth assessment of similarities and differences, and variability related to different facets of AD management. The content analysis also captured key differences, if any, between specialties.

## 3 RESULTS

### 3.1 Characteristics of participating PCPs

**FIGURE 2:**
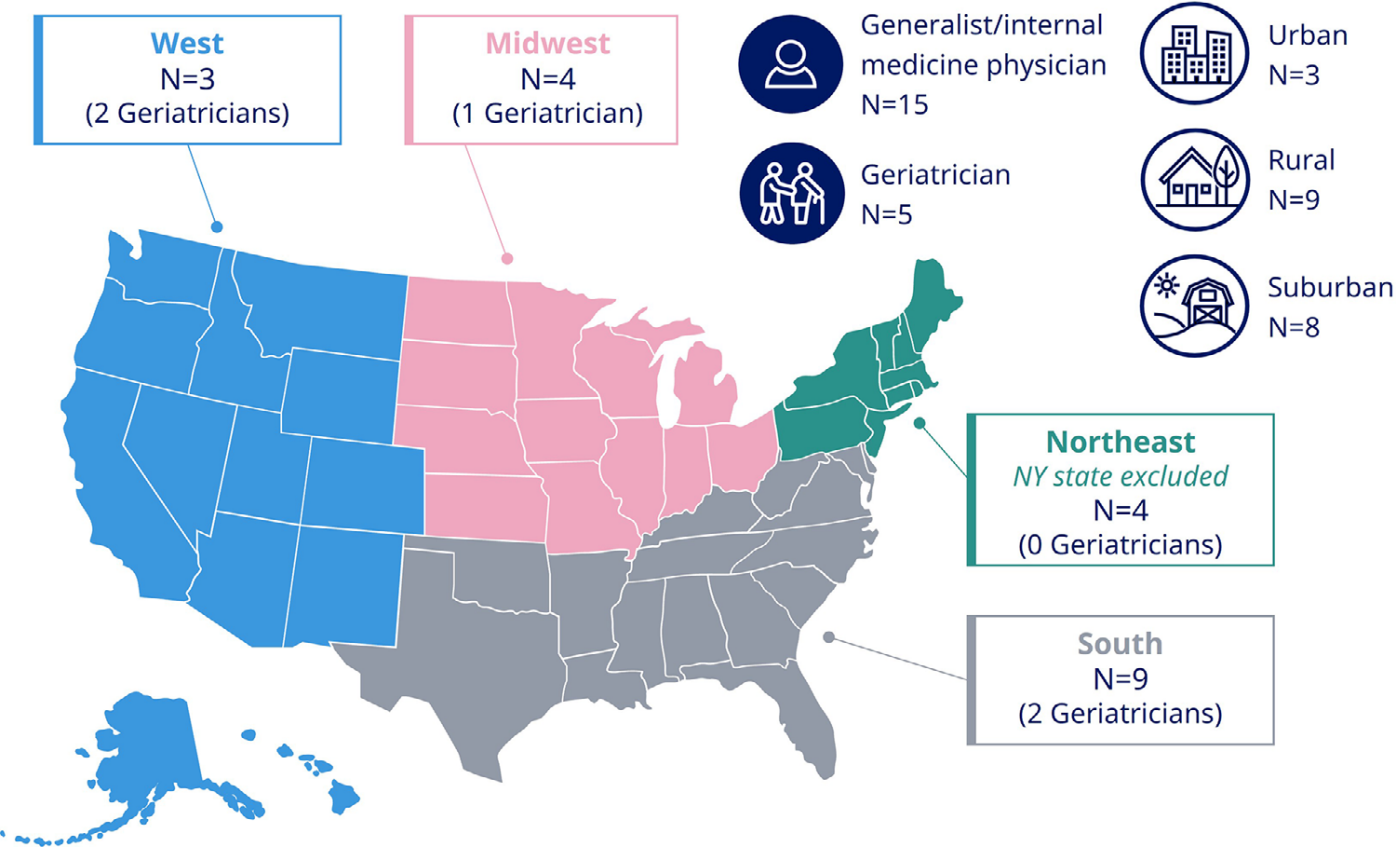
Characteristics of participating PCPs. N, number of participants; PCP, primary care physician.

The 20 PCPs who were interviewed were geographically distributed across the United States, representing urban (*N* = 3), rural (*N* = 9), and suburban (*N* = 8) locations and consisting of both generalists (*N* = 15) and geriatricians (*N* = 5) as summarized in **Figure** **2**.

### 3.2 Responses to provocative statements and summarized findings

**FIGURE 3:**
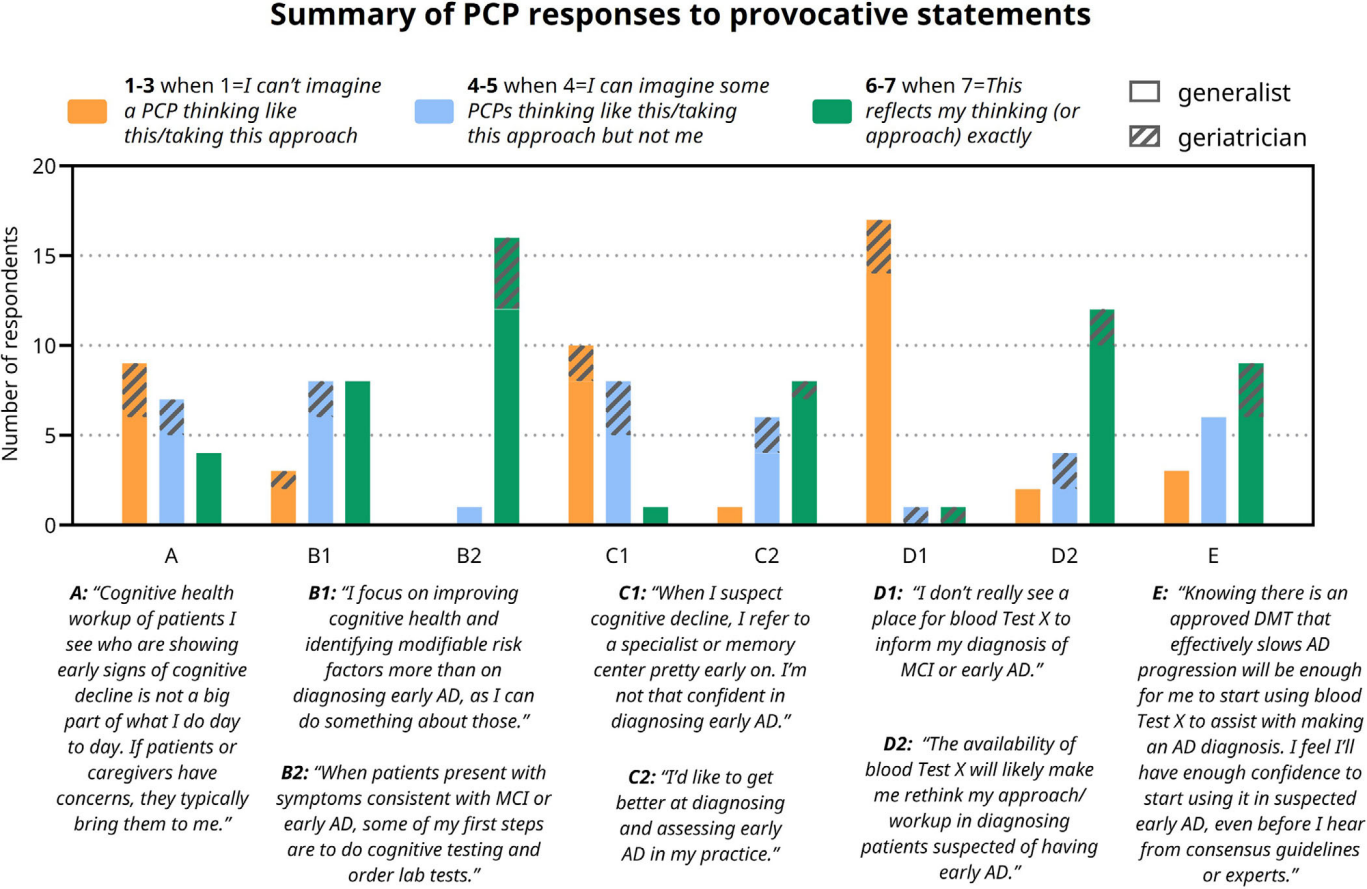
Summary of PCP responses to provocative statements. Respondents were allowed to skip statements. AD, Alzheimer's disease; DMT, disease‐modifying therapy; MCI, mild cognitive impairment; PCP, primary care physician.

The PCPs’ responses to the provocative statements are collected in **Figure** **3**, and findings are summarized in Sections 3.2.1–4 by current PCP role, perceptions, and practice in AD; current PCP barriers to AD diagnosis; PCP reaction to BBM tests; and potential impact of use of BBM tests.

#### 3.2.1 Current PCP role, perceptions, and practice in AD

Most PCPs reported that investigation of cognitive decline is an important part of their role. PCPs also reported that AD diagnosis is important to determine the most appropriate treatment and help affected individuals and their families to access resources and help plan for the future. PCPs claim to be somewhat confident in diagnosing and staging AD but would like to improve their skills and resources. They currently see many unmet needs, having to conduct a complex and lengthy workup with no definitive diagnostic tests or treatments with a risk–benefit profile suitable for primary care use.

#### 3.2.2 Current PCP barriers to AD diagnosis

PCPs reiterated previously identified barriers. These included stigma of AD due to poor prognosis and heavy disease burden, complexity and time‐consuming nature of current diagnosis, diagnosis by exclusion (ruling out other potential causes of cognitive decline) and lack of easy diagnostic tools, lack of effective treatments to change the disease course, and the PCPs’ preference to manage comorbidities and lifestyle. Hypothesized barriers that were found to be less important or did not resonate included PCPs’ lack of understanding or awareness of MCI and AD, lack of agreement on priorities between affected individual, family, and clinician, as well as lack of access to specialists to assist in diagnosis.

#### 3.2.3 PCP reaction to BBM tests

Using the hypothetical Test X as an example, the PCPs’ response to BBM tests was positive, with almost all (17 of 19 responses to Statement D1) seeing a role for them in their practice. Generalists were more positive regarding diagnostic BBM tests than geriatricians, who saw less benefit in relation to their current tools and skills. Perceived key strengths of BBM tests were the reported sensitivity and specificity, simplicity of conduct, and likely ease of access or lower health care system cost. Negative reactions were few and included BBM tests not being definitive but rather additive to current practice. PCPs also sought clarity on where to implement BBM testing in the diagnostic pathway, that is, when in the course of a cognitive evaluation should such tests be performed: simultaneously with existing clinical assessment methods (cognitive testing, standard lab workup); as a second stage, after clinical assessment methods but before traditional assessments for confirmation of AD pathology (i.e., before PET scan or CSF testing); or as a replacement of traditional amyloid confirmation methods (i.e., PET scan or CSF testing).

#### 3.2.4 Potential impact of use of BBM tests

Assuming BBM testing becomes accessible and affordable, most PCPs would like to order the tests early, that is, while or soon after ruling out key modifiable causes of cognitive decline. There were differing opinions about the timing of integration into clinical care pathways for individuals with symptoms of cognitive decline. Key identified benefits of BBM tests included: more confidence in diagnosing early AD, reassurance for the individual with AD and their family about accurate diagnosis, helping individuals with AD and their families acknowledge the situation and start planning for the future, and giving the PCP confidence to initiate treatment early, despite very limited DMT options. It should be noted that aducanumab was the only DMT approved for clinical use in the United States at the time of these interviews and the uptake was very low. Key barriers for implementation of BBM testing included possible cost or insurance issues requiring testing later in the diagnostic sequence than the PCPs would like.

## 4 DISCUSSION

The PCPs interviewed in our study believe that investigating cognitive decline is an important part of their role. Although they feel somewhat confident in diagnosing and staging AD, they would like to improve their skills. Almost all of the PCPs interviewed responded positively to the idea of BBM tests and saw a role for BBM tests in their practice, potentially giving them more confidence in diagnosing AD early and initiating treatment. Increased diagnostic confidence and saved time could empower PCPs to meet the growing need for early AD diagnosis.

Clinical practice guidelines specific for primary care have been published to provide a structured framework for evaluating individuals with suspected AD.^17^ The guidelines note that as BBMs become more accurate and validated in real‐world settings, these guidelines will require updating to incorporate their use. BBM tests have the potential to help physicians make more definitive AD diagnoses and ensure appropriate individuals gain access to DMTs.^17^ However, the guidelines note that availability and reimbursement terms for these tests remain unresolved. The corresponding specialty care guidelines^23^ take a cautiously optimistic stance on BBM tests but do not yet recommend them as an alternative to traditional amyloid PET or CSF biomarker assessments.

Although two DMTs approved to treat early AD are currently available in the United States, neither was available when the interviews for this study were conducted.^24^, ^25^ More DMTs are expected to enter the market with increased clinical use in the coming years.^26^ New DMTs are likely to increase interest in and motivation for early AD diagnosis and thereby the potential employment of BBM tests, as supported by approximately half of the PCPs interviewed in this study. Furthermore, the availability of BBM testing can in turn drive the development of new DMTs through easier recruitment and more relevant cohorts for clinical studies.^26^

As more individuals seek help with cognitive decline, the involvement of the primary care sector is becoming crucial^16^, ^17^ and could be supported by the availability of BBM testing.^27^ The Global CEO Initiative on Alzheimer's Disease (CEOi) is working to bring BBM tests into clinical practice, especially in primary care. For primary care use, the CEOi recommends a sensitivity of ≥90% and specificity of ≥85% for use as a triaging test that is followed up by a confirmatory test.^28^ Although the current study was conducted before the CEOi recommendation was published, the hypothetical Test X presented to the participating PCPs had a 91% sensitivity and 86% specificity, and such a test was viewed very positively. Although these PCPs had a favorable attitude toward BBM tests in their practice, not all prior studies have found the same.^29^

An interdisciplinary geriatrics summit provided recommendations to improve early detection of cognitive impairment in older adults in primary care, reinforcing the benefits of a multidisciplinary approach, including PCPs and specialists.^30^ Results from the current study show that PCPs have the interest and motivation to increase accurate diagnosis of early AD, and availability of BBM testing could give them the confidence to do that. Of note, an integrated workflow can make timely diagnoses more likely, thus improving access to care for affected individuals.

The PCPs interviewed in this study were generally very positive about implementation of BBM tests in primary care. Their concerns related to insurance coverage as well as a need for clear guidelines on where BBM tests fit in the diagnostic pathway and on the interpretation of test results. Recent recommendations and considerations for clinical implementation of BBM tests for AD recognize the same challenges.^21^, ^31^, ^32^, ^33^ Several initiatives are ongoing to facilitate implementation of BBM testing in the AD diagnostic workflow, such as the Davos Alzheimer's Collaborative (DAC) Accurate Diagnosis project, Clinical Implementation of Amyloid Neurodegeneration and Tau testing in Primary Care (CANTATE‐PC), the AD‐RIDDLE consortium in Europe, and the REAL AD study in Sweden. The ongoing initiatives will support health care system readiness as the first BBM tests become widely available for use in primary care.

### 4.1 Limitations

The exploratory nature of this study and the small sample size of 20 PCPs may limit the generalizability of the findings.

### 4.2 Conclusion

Our study highlights PCPs’ attitudes, perceptions, barriers, and receptivity toward BBM tests for early AD diagnosis in primary care settings. PCPs had a positive response to incorporating BBM tests into their practice, with the majority reporting that evaluating causes of cognitive decline is an important part of their role and that AD diagnosis is essential to determine the most appropriate treatment. This provides evidence, from the PCP perspective, that BBM tests are well positioned to streamline diagnostic workflows and support earlier, more confident detection of AD in the primary care setting.

## AUTHOR CONTRIBUTIONS

Data were analyzed by C2N Diagnostics. All authors contributed to interpretation of data as well as writing, reviewing, and editing of the manuscript. All authors approved the final version of the manuscript.

## CONFLICT OF INTEREST STATEMENT

Jeffrey M. Burns has received research support from the National Institutes of Health (NIH), Gates Ventures, and Hyperfine, Inc; research support to conduct clinical trials (paid to institution) from Eli Lilly, Biogen, Eisai, AbbVie, Astra‐Zeneca, Roche, and Ionis; has served as a consultant for Renew Research, Eisai, Eli Lilly, Labcorp, Roche, Renew Biotechnologies, Abbvie, and Novo Nordisk; and serves on a Data Monitoring Committee for Intra‐Cellular Therapies, Inc.

Susan Alford has received personal compensation for serving as an employee of Novo Nordisk and has stock in Novo Nordisk.

Justine Coppinger has received compensation in the form of salary as an employee of C2N Diagnostics.

Martí Jiménez‐Mausbach has received personal compensation for serving as an employee of Novo Nordisk and has stock in Novo Nordisk.

Sutapa Ray has received compensation in the form of salary as an employee of C2N Diagnostics.

Hemant Pandey has received research support to conduct clinical trials (paid to institution) from Eli Lilly and has served as a consultant and speaker for Eli Lilly, Eisai, and Novo Nordisk.

Rosemary Laird has received research support to conduct clinical trials (paid to institution) from Eli Lilly, Biogen, Eisai, and AbbVie. Author disclosures are available in the Supporting Information.

## CONSENT STATEMENT

All primary care physicians who were interviewed for this study provided informed consent.

## Supporting information



Supporting Information

Supporting Information

Supporting Information

Supporting Information

Supporting Information
