# Supplementary material for: Early Alzheimer's diagnosis: U.S. primary care physicians and use of blood biomarkers
Source: Alzheimers Dement. 2026 Jan 18;22(1):e70986. doi: 10.1002/alz.70986 (PMC12812852; doi:10.1002/alz.70986)
Supplement: Supplementary file 1 — Supporting Information [file ALZ-22-e70986-s005.docx]

# Early Alzheimer’s diagnosis: US primary care physicians and use of blood biomarkers

Jeffrey M. Burns, Susan Alford, Justine Coppinger, Martí Jiménez-Mausbach, Sutapa Ray, Hemant Pandey, Rosemary Laird

Supplementary Table 1: **Test X characteristics**

| Test Description | - **Test X** is a **blood** test developed to aid in the diagnosis of **Alzheimer's disease (AD) for patients aged 55 and older who present with cognitive impairment and are being evaluated for AD and other causes of cognitive decline** - Test X measures the **concentration of multiple analytes in the plasma to evaluate for presence of brain amyloid plaques**   - Analytes include amyloid beta peptides, tau peptides   - Technology used: mass spectrometry - Test X combines the analyte measurements into a **clinically validated algorithm** to determine a patient’s likelihood for having a positive amyloid PET scan |
| --- | --- |
| Test Result & Turnaround Time | - Test X result output is a numeric score (0-100), which assigns likelihood for presence of brain amyloid pathology as **Negative** or **Positive** - 2 weeks |
| Test Interpretation | - A **Negative** result score (0-47) is consistent with a negative amyloid PET scan, reflecting a low likelihood of amyloid plaques - A **Positive** result score (48-100) is consistent with a positive amyloid PET scan, reflecting a high likelihood of amyloid plaques - As currently designed, Test X is **NOT intended as a stand-alone diagnostic assay** [currently there is no test that is stand-alone for AD with the exception of post-mortem examination] - **Test X results are** **interpreted in the context of other information about the patient**, such as medical and family history, neurological examination, neurobehavioral tests, imaging, and routine laboratory tests |
| Diagnostic Accuracy | - Positive percent agreement (PPA) or sensitivity^a^ 91% - Negative percent agreement (NPA) or specificity^b^ 86% - Accuracy 89% - Positive Predictive Value 91% - Negative Predictive Value 86% |

^a^Sensitivity: ability of a test to correctly identify those with disease (true positive)

^b^Specificity: ability of a test to correctly identify those without the disease (true negative)
