# Supplementary material for: Early Alzheimer's diagnosis: U.S. primary care physicians and use of blood biomarkers
Source: Alzheimers Dement. 2026 Jan 18;22(1):e70986. doi: 10.1002/alz.70986 (PMC12812852; doi:10.1002/alz.70986)
Supplement: Supplementary file 2 — Supporting Information [file ALZ-22-e70986-s003.pdf]

Early Alzheimer's diagnosis: US primary care physicians and use of blood biomarkers

Jeffrey M. Burns[1], Susan Alford[2], Justine Coppinger[3], Martí Jiménez Mausbach[4], Sutapa Ray[3], Hemant Pandey[5], Rosemary Laird[6,7]

[1]University of Kansas Alzheimer's Disease Research Center, Kansas City, KS, USA; [2]Novo Nordisk Inc., Plainsboro, NJ, USA; [3]C2N Diagnostics LLC, Saint Louis, MO, USA; [4]Novo Nordisk A/S, Soeborg, Denmark; [5]Brain and Spine Center, Chandler, AZ, USA; [6]Mymemoryclinic.org, Melbourne, FL, USA; [7]NAN Navigator Inc., Orlando, FL, USA.

What is the study about?

- Investigate how primary care physicians (PCPs) view their role in diagnosing Alzheimer's disease (AD).
- Find out PCPs' opinions on using blood biomarker (BBM) tests in AD diagnosis when they become available.

Traditional diagnostic methods are slow, complex, and costly.

BBM tests offer a simpler and more accessible alternative for early AD diagnosis.

What was done?

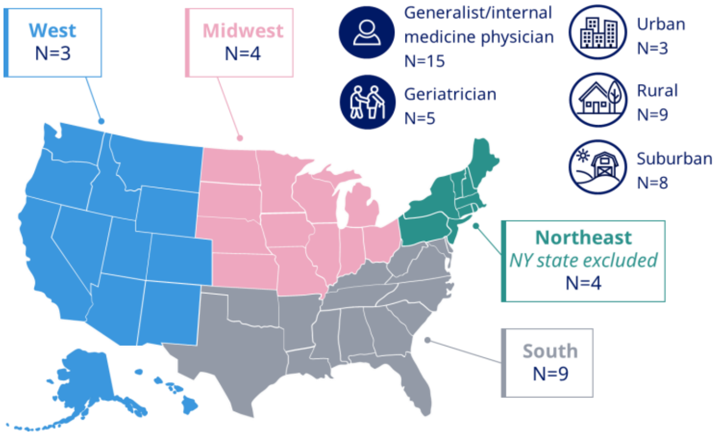

20 PCPs across US

60-minute in-depth online interview

Topics

AD diagnosis
PCP roles
BBM tests
Obstacles

Analysis and summary of interviews

What were the results?

Most PCPs believe investigating cognitive decline is an important part of their role and they feel somewhat confident in diagnosing AD.

PCPs face barriers such as the complexity of current diagnostic methods and the stigma of AD diagnosis.

PCPs responded positively to BBM tests, viewing them as accurate and cost-effective.

Potential barriers for implementation of BBM testing include lack of education on interpretation and implementation, reimbursement and lack of clarity on their placement in the diagnostic pathway.

What were the conclusions?

- Majority of PCPs reported that evaluating causes of cognitive decline is an important part of their role and had a positive response to incorporating BBM tests into their practice.
- From the PCP perspective, BBM tests appear well positioned to streamline diagnostic workflows and support earlier, more confident recognition of AD in the primary-care setting.
- Consensus guidelines on AD diagnosis and education on use and interpretation of BBM tests may benefit their implementation in primary care.
