# Supplementary material for: Early Alzheimer's diagnosis: U.S. primary care physicians and use of blood biomarkers
Source: Alzheimers Dement. 2026 Jan 18;22(1):e70986. doi: 10.1002/alz.70986 (PMC12812852; doi:10.1002/alz.70986)
Supplement: Supplementary file 3 — Supporting Information [file ALZ-22-e70986-s001.docx]

# Early Alzheimer’s diagnosis: US primary care physicians and use of blood biomarkers

Jeffrey M. Burns, Susan Alford, Justine Coppinger, Martí Jiménez-Mausbach, Sutapa Ray, Hemant Pandey, Rosemary Laird

## Lay language summary: Understanding primary care physicians’ opinions on early Alzheimer's disease diagnosis and the potential use of blood biomarker tests

**Simple blood tests may soon be used to assist with Alzheimer’s disease (AD) diagnosis.**

- Early and accurate diagnosis of AD is crucial for initiating treatment at an earlier stage of disease.
- Traditional diagnostic tools are slow, complex, and costly.
- Blood biomarker (BBM) tests offer a simpler and more accessible alternative to aid in early AD diagnosis.

**This study sought to understand primary care physicians’ (PCP) opinions on early AD diagnosis and the potential use of BBM tests.**

- A total of 20 PCPs in the US were interviewed for 60 minutes.
- The interview focused on different topics: PCP’s role in AD diagnosis, BBM diagnostic tests, and obstacles to implementation.

**PCPs are interested in using BBM tests for early AD diagnosis but need more knowledge on when and how to use them.**

- Most PCPs believe that they play an important role in investigating memory loss and are somewhat confident in diagnosing AD.
- PCPs face barriers such as the complicated diagnostic workflow and negative perceptions of an AD diagnosis.
- PCPs responded positively to BBM tests, viewing them as accurate and cost-effective.
- Concerns included insurance coverage for BBM test use and clarity on when these tests should be used and how the results should be interpreted.

**PCPs think that BBM tests could help to diagnose AD early and secure appropriate care.**
