# Supplementary material for: Early Alzheimer's diagnosis: U.S. primary care physicians and use of blood biomarkers
Source: Alzheimers Dement. 2026 Jan 18;22(1):e70986. doi: 10.1002/alz.70986 (PMC12812852; doi:10.1002/alz.70986)
Supplement: Supplementary file 4 — Supporting Information [file ALZ-22-e70986-s004.docx]

**DISCUSSION GUIDE**

**Respondent introduction (2 min)**

Moderator: *“To start, I had some questions about you and your medical practice.”*

1. Please tell me a little about your practice:
   - Region of the US
   - Sub-specialty or focus if any, e.g., care of elderly
   - Years in practice
   - Practice setting(s)
     - Solo, single specialty or multigroup practice
     - Whether independent or part of system or network
     - If part of hospital system: academic or not?
   - General details about your patient population
   - Any care home or nursing home responsibility?
2. How many patients do you see in a typical week?
   - How many patients do you see in a typical week with signs and symptoms of mild cognitive impairment (MCI) or any type or stage of dementia? **Moderator clarify if needed:** Please include early Alzheimer’s Disease and others at a mid to late stage of dementia?
   - In what settings do you see these patients?
   - What are the typical reasons for visits with these patients? **Allow unaided response, then probe as needed:** focus on MCI / dementia management versus other reasons e.g., care of concomitant conditions?
   - Who else provides care for these patients at your practice (e.g. NP / PAs)? **If others:** How are your roles defined?

**Respondent high-level knowledge of and role in early AD (5 min)**

Moderator: *“Let’s talk a bit more about visits and consults you have with patients with signs or symptoms of cognitive impairment or early Alzheimer’s dementia (AD). Just to clarify by ‘patients with early AD’, for the purposes of this interview I mean patients showing signs and symptoms of AD at an early stage rather than ‘early onset dementia’.”*

1. Thinking of these patients in your practice, please describe how you see your role in identifying possible causes of the patient’s cognitive impairment? **Allow unaided response; probe as needed:** To what extent do seek to establish a specific cause of the cognitive impairment?
   - **If ‘establish specific cause’ to some extent:** What causes do typically try to rule in or rule out?
   - At what point do you consider relying on a “clinical diagnosis”?
   - How do you do this? What kind of specific evaluation do you pursue to achieve your final diagnosis?
2. Next I’d like to do a ‘quick fire’ exercise: please tell me 3 words that come to your mind when I say: **“early stage Alzheimer’s Disease”**. **Allow time**. Why do those words come to your mind?
   - **Probe as needed:** To what extent are you familiar with AD staging – or is it just “AD” as far as you are concerned when you’re dealing with these patients?
3. What does “*early* stage AD” mean to you?
   - **Probe:** To what extent do you distinguish stages of Alzheimer’s Disease in your day-to-day practice?
   - Do *you* get involved in AD staging? In what way? Please describe what you do to identify if it’s an *early* stage of AD.

**DMT awareness, understanding, and future-framing (5 min)**

Moderator: *“Let’s now move on to talk about potential treatments for AD.”*

1. **Refer to screener rating at S7a (*“how often seek to keep up to date on AD developments”*)** When we asked you in the earlier recruitment survey about how often you seek to keep yourself up to date on AD drugs in development you said [**Moderator give rating**]. Please tell me more about that.
2. What is your understanding of a “Disease Modifying Therapy” or DMT for AD? What current AD drugs do you consider to modify or slow disease progression, if any? **Listen for and probe respondent understanding if symptomatic treatments mentioned**
3. Are you aware of any new drugs or compounds in development that modify AD? **If yes:**  which products? What are you interested in? Why?
4. **If ‘no’ or respondent mindset about potential DMTs unclear, refer to screener rating at S7b (*“how optimistic about DMTs to slow disease progression in early AD”*)** When we asked you in the earlier recruitment survey about how optimistic you are about the prospect of DMTs to slow progression in early AD you said [**Moderator give rating**]. Please tell me more about that.
5. **Ask all:** What in your view would constitute a clinically meaningful outcome for a drug that modifies or slows disease progression in AD?
   - **Probe as needed:** The patient stays at the same stage of disease for longer? The patient’s memory does not decline further? Other outcome?

For the balance of our discussion, please assume we are in a world where there is at least one DMT available that slows the progression of cognitive decline in early AD.

**Statement Reactions (pre-BBBM information) (~20 mins)**

Moderator: *“Next I’d like to get your perspective on some findings we’ve had when discussing these and related topics with physicians in other research.”*

Moderator say: *In the course of previous research we have heard various points of view on the challenges of identifying and diagnosing patients with MCI or early AD. I’d like to show you different points of view we uncovered during this research to understand if you identify with it in any way, as well as your thoughts on what may be reasons why your peers may have this point of view.*

**Moderator to expose each statement in rotation (hiding others) to get agreement rating, discuss, address related probes, before moving on to next statement**

1. How would you describe your **initial reaction** to [STATEMENT code]? **Allow unaided response.**
   - **Use Rating Showcard.** How would you rate this statement on this scale from 1 to 7 where…
   - 1 is “*I can’t imagine a PCP thinking like this / encountering this issue”*
   - 4 (middle of the scale) is: “*I can imagine some PCPs thinking like this / encountering this issue, but not me”*
   - 7 is: “*This reflects my thinking, or experience / I encounter exactly this issue”*
   - Why is your rating [X]? To what extent do you share this point of view?
   - What, ultimately, is behind PCPs thinking in this way?
   - (**Each barrier / belief mentioned**) What is needed to address this problem?
2. **Moderator: ask statement-specific probes below, adjusting according to what discussed unaided in above discussion of rating**

Identification and early AD priority / PCP hesitancy

***A1: “Cognitive health work-up of patients I see who are showing early signs of cognitive decline is not a big part of what I do day to day. If patients or caregivers have concerns, they typically bring them to me.”***

**If high rating (agree):**

- What are the reasons for early cognitive health work-up being a relatively low priority for you / your colleagues? **Listen for / probe if not mentioned:** Time? Expertise? Sensitive subject to address? Other? Mix of these – if yes, what is the chief reason?
- When, if at all, would you proactively address cognitive symptoms, even if the patient or their family doesn’t bring this to your attention?
- What might facilitate a more proactive approach for you?
- In terms of who “pushes” for a diagnosis, how does this change as symptoms progress? Does the PCP play a more active role in diagnosing AD with more severe or advanced symptoms? When / why / why not?

**If low rating (disagree – i.e., 5 or less):**

- What causes you to suspect or be concerned about cognitive issues? **Probe as needed:**
  - What is the trigger for you to address and conduct work-up for cognitive symptoms in the absence of patients or caregivers themselves bringing this to your attention?
  - Can you give me examples of when you decide to broach cognitive health work-up with a patient or their family even if they are not pushing to address this?
- What, ultimately, drives you to do this? What is your purpose?
- What is your approach to early cognitive health work-up?
- How have you developed this approach? What is different about your practice compared to practices / practitioners who don’t give priority to assessing early signs of cognitive decline?

**Any rating (if not addressed above):** How persistent are patients and their family members in pursuing confirmation of a diagnosis of AD or other dementia? Why is this important? Is this different in early AD compared to when symptoms are more advanced? How? Why?

- - Who plays the more prominent role – you, the patient or their family etc. – in pursuing an AD diagnosis as the disease progresses?

I’d like to explore possible emotional barriers in assessing cognitive decline in patients…

- To what extent are there emotional barriers for the patient and family to raise the issue, or are they simply unaware? Why is this? **Probe as needed:** Fear of diagnosis? Fear about the future? Denial?
- To what extent are there emotional barriers for the physician to raise the issue? Why is this? What are physicians’ concerns?
- What is an appropriate way for you as the PCP to proactively address cognitive symptoms that may be a sign of early AD?
- Is there anything that could facilitate a more proactive approach for colleagues who are aligned with with this statement?

1. Initiating diagnosis

***B1: “I focus on improving cognitive health and identifying modifiable risk factors more than on diagnosing early Alzheimer’s disease (AD), as I can do something about those.”***

**If high rating (agree):**

- Why is that? What do you do?
- What is the benefit of giving priority to “optimizing cognitive health and identifying modifiable risk factors”?
- What are the concerns or risks of pursuing a diagnosis of early AD in patients with signs of cognitive decline?
- What would facilitate you or colleagues giving a higher priority to diagnosing early AD? **Listen for “arrival of treatments to address AD disease progression”. If mentioned:** Is anything else needed to facilitate such a change in approach?

**If low rating (disagree):**

- Why is that? What do you do?
- What are the benefits of pursuing a diagnosis in patients with signs of cognitive decline that are consistent with early AD?

***B2: “When patients present with symptoms consistent with mild cognitive impairment (MCI) or early AD, some of my first steps are to do cognitive testing and order lab tests.”***

**If high rating (agree):**

- How confident are you in your next steps when you see patients with signs of MCI or early AD?
- What do you do? **Probe:**
  - Cognitive test in the office? Which – MMSE, MoCA, other? How confident are you in doing these?
  - Which lab tests do you order, and how quickly after you first identify signs of MCI? What is the purpose of each test?
  - **If imaging tests (CT scan / MRI) mentioned:** In terms of interpretation, what is your role vs that of the radiologist?
  - How does the clinical context impact the way you make the diagnosis – or the tests used to aid your determination – i.e. if there is a “high or low clinical suspicion” of AD?

**If low rating (disagree):**

- Why is that? What do you do? **If refer to specialist, see probes below**
- For patients with signs of MCI or eAD, what do you feel is needed for PCPs to move more quickly to ordering lab tests or do cognitive testing?

**Any rating:**

- What, if any, challenges do you encounter ordering blood tests? How routinely do you order these? **If not routine:**  Why is this not done for all patients?
- What is the purpose of [blood tests mentioned]? **Listen for: excluding causes other than AD**

1. PCP role and referral

***C1: “When I suspect cognitive decline, I refer to a specialist / memory center pretty early on. I’m not that confident in diagnosing early AD.”***

**If high rating (agree):**

- What triggers you to refer to a specialist or memory center / Center of Excellence (COE)? How long does it take you to do this?
- Why is this your preferred approach?
- What would facilitate you to take steps toward investigating underlying causes of the cognitive issues prior to referral?

**If low rating (disagree):**

- Why is that? What do you do prior to specialist referral? **Explore steps taken in work-up if not addressed in (B) above.**
- To what extent do you rely on guidelines to support your practice today? Which? How helpful are they? What is missing?

***C2: “I’d like to get better at diagnosing and assessing early AD in my practice.”***

**If high rating (agree):**

- Why? What would be the benefit of playing a greater role in ***diagnosing and assessing early AD***?
- What have been the barriers in the past (to getting better at diagnosing and assessing early AD)?
- What is needed to help you?

**If low rating (disagree):**

- Why is that? What are the barriers to PCPs playing a greater role in this regard? **Probe as needed:**
  - Are you aware of any guidelines to support PCPs in the assessment of symptoms that are consistent with early AD?
  - Is it a question of lack of appropriate guidelines – or do barriers lie elsewhere?
    - **Prompt if not mentioned:**  how comfortable are you doing cognitive assessments as part of work-up?

1. **After reviewing and discussing statements so far:** Thinking about challenges PCPs may face in putting eAD at a higher priority, what are the attitudes and experiences that resonate most /?
   - What other challenges now come to mind – that maybe you don’t experience but others might?

**BBM information and reaction to remaining statements (10-15 mins)**

Moderator: *“Before we continue our discussion, I’d like to share with you some information about a hypothetical blood test. For the purposes of discussion, please assume this test actually becomes available for physicians to use.”*

**[PLACEHOLDER: show stimulus on “Blood Test X”, and sample report on screen]**

1. What is your reaction to this information [positive / negative / neutral]. Why do you say that?
   - How do you see this fitting into your current practice [if relevant] for assessing patients with symptoms of cognitive decline?

Moderator: *“Now with this context in mind, let’s continue.”*

**PLACEHOLDER: repeat above process for remaining statements:**

D: Adoption of “*Blood Test X”*

***D1: “I don’t really see a place for Blood Test X to inform my diagnosis of mild cognitive impairment (MCI) or early AD.”***

**If high rating (agree):**

- Why don’t you see a place for Blood Test X in your practice? **Probe as needed:**
  - To what extent is this a matter of: unclear benefit; lack of priority for you as the PCP; logistical barriers; other reasons?
- What would facilitate you or colleagues integrating Blood Test X into your practice? **Probe:**
  - Inclusion in guidelines? Specialist endorsement? Reimbursement? Other?

**If low rating (disagree):**

- Why is that?
- Where would Blood Test X fit into your existing workup of patients?
  - Where does it come in the sequence of steps in assessing patients?
  - What, if any, steps would you still take before ordering Blood Test X?
  - Who else would be involved in your practice - e.g., NP / PA?
  - Depending on the result, what do you, as PCP, do next?

***D2: “The availability of Blood Test X will likely make me rethink my approach/workup in diagnosing patients suspected of having early AD.”***

**If high rating (agree):**

- Please talk me through how you see Blood Test X changing your approach and role with regard to these patients. **Probe as needed:**
  - What do you see as the primary purpose or benefit of adopting Blood Test X?
  - To what extent do you see Blood Test X as an aid to ***your*** diagnosis of early AD, or as a way to triage patients for Specialist referral? Please talk me through your thinking.

**If low rating (disagree):**

- Why is that?
- Why do you think your current approach to workup is adequate without Blood Test X?
- What else would be needed for you to change your approach?

E: DMT as Primary Driver

***E1: “Knowing there is an approved disease-modifying treatment (DMT) that effectively slows AD progression will be enough for me to start using Blood Test X to assist with making an AD diagnosis. I feel I’ll have enough confidence to start using it in suspected early AD, even before I hear from consensus guidelines or experts.”***

**Any rating (explore reasons why, then probe):**

- In a future with Blood Test X that can help accurately diagnose patients with AD (alongside cognitive tests and other assessments to exclude other causes), and an approved DMT that effectively slows AD progression, please talk me through how you see your role as PCP in diagning and managing patients at an **early** stage of disease?
- How different (or not) is this role from today? **If changed:** what is the key driver of change – the existence of a DMT; something you have learned about Blood Test X; evolving guidelines; other?

**If high rating (agree):**

- Please talk me through your reasons: what will be the benefit of rapidly adopting Blood Test X into your practice when you suspect early AD?

**If low rating (disagree):**

- Why is that?
- What do you need to hear from guidelines or experts?

**After all statements reviewed, moderator shows all on screen.**

1. Now that we’ve reviewed all these statements and thought about the challenges relating to each, which statements and their related challenges do you think are going to be most relevant for PCPs to adopt a blood test that can help diagnose early AD?
   - **If availability / access of test or DMT prioritized:** what else is needed for PCPs to have greater involvement in diagnosing early AD?
   - **If multiple challenges identified:** Thinking about what needs to change for PCPs like yourself to adopt Blood Test X more readily, how would you rank these challenges in order of importance?

**Implementing blood test in practice (5 mins)**

Moderator: “*I’d like for you to consider what is needed for you and your peers to adopt the “Blood Test X” into your day-to-day practice once this is available”*

1. What or who would influence your prescribing practices for “Blood Test X”?
   - **Probe to identify top 3 influencing factors.**
   - Through which channels would you most like to learn about it? **Allow unaided response, then probe for importance of:**
     - Professional societies – which? (E.g., American Academy of Neurology / AAN)
     - Guidelines
     - Manufacturer communications
     - Journals
     - Online resources e.g. Medscape, Up-to-Date etc.
     - (Dinner) meetings with local medical colleagues who have started using the Test
   - Can you think of other tools or resources that would increase your motivation to adopt the test?

**Finally, please assume you are aware of the test and the communication is such that you are now ready to use it in your own practice.**

1. What do you perceive would be the biggest barrier to you ordering “Blood Test X” in your day-to-day practice?
   - How motivated are you to address [**moderator vocalize stated barrier**] - and any other barriers?
   - What could close the gap to enable you to order the test when you feel it is appropriate?
2. Please talk me through how you would change the way you evaluate patients with issues of cognitive impairment, if at all. **Moderator – circle back to how respondent described their current approach:** How much of a change does this represent compared with what you described earlier?
   - What are the steps to integrate Blood Test X into your practice?
   - **For each step:** Who else is involved? What is their role?
     - How easy or difficult is this step? What would help you overcome any barriers? **Probe if not mentioned:** do you see any specific barriers relating to: administering the test? Reading the results?
   - What role will access and cost play? Please walk me through the scenarios you envisage.
   - What role will practice staff play in supporting implementation of Blood Test X? Who in your practice will be involved?
   - What could help you overcome these barriers?

**Moderator: check for any further questions**

***“Thank you so much for your time today!”***
